# Anti-Inflammatory Effects of *Sosiho*-Tang, a Traditional Herbal Formula, on Acute Lung Injury in LPS-Sensitized Mice and -Raw 264.7 Cells

**DOI:** 10.1155/2021/6641689

**Published:** 2021-02-09

**Authors:** La Yoon Choi, Mi Hye Kim, Da-Hwa Jung, Woong Mo Yang

**Affiliations:** ^1^Department of Convergence Korean Medical Science, College of Korean Medicine, Kyung Hee University, Seoul 02447, Republic of Korea; ^2^National Development Institute of Korean Medicine, 288 Udeurandeu-gil, Sejong, Janheung-gun 59338, Republic of Korea

## Abstract

Acute lung injury (ALI) is a series of syndromes with persistent inflammation and abnormally increased vascular permeability. *Sosiho*-tang (SSHT), a traditional herbal formula consisting of a mixture of seven herbs, has been used to treat allergic reactions and chronic hepatitis disease in East Asia. In this study, we determined whether SSHT has an inhibitory effect against lipopolysaccharide- (LPS-) induced acute lung injury (ALI) in mice. 0.05, 0.55, and 5.55 mg/kg of SSHT were orally administered to C57BL/6J mice for 7 days prior to the administration of LPS. After 2 h of LPS sensitization, lung tissues were collected to confirm the lung histology and ALI-related inflammatory factors. SSHT ameliorated the LPS-induced alveolar hemorrhage, alveolar wall thickening, and the shrinkage of the alveolar spaces in the ALI mice model. Proinflammatory cytokines including IL-6, TNF-*α*, and IFN-*γ* in the lung tissue were significantly regulated in the SSHT-treated groups compared to the LPS only-treated group. Also, increases of IL-6 and TNF-*α* and decrease of IFN-*γ* expressions were dose-dependently modulated by SSHT treatment in LPS-induced raw 264.7 cells. Additionally, the translocation of NF-*κ*B into nucleus and phosphorylation of mitogen-activated protein (MAP) kinase were significantly attenuated by the treatment of SSHT in LPS-sensitized ALI mice. SSHT showed anti-inflammatory activities by inhibiting proinflammatory cytokines and NF-*κ*B signaling in LPS-induced ALI. This study demonstrates that SSHT has preventive effects on LPS-induced ALI by regulating inflammatory responses as an alternative for treating lung diseases.

## 1. Introduction

Acute lung injury (ALI) is a life-threatening medical complication with a range from 52 to 65% of mortality rate in patients [1]. ALI is associated with sepsis, traumatic injuries, inhalation, massive blood transfusion, bilateral pulmonary infiltration, and hypoxemia [2]. It is commonly accompanied by systemic inflammatory responses following pulmonary edema, increase of alveolar capillary barrier permeability, and excessive cytokines production in lung [3–5]. In the previous studies, lipopolysaccharide (LPS), component of Gram-negative bacteria, has been known to induce the release of proinflammatory cytokines such as tumor necrosis factor-*α* (TNF-*α*) and interleukin-6 (IL-6), which stimulates alveolar hemorrhage, alveolar wall thickening, and alveolar spaces shrinkage [6, 7].

Clinically, treatment of ALI focuses on counterbalance of inflammatory response by regulating inflammatory/anti-inflammatory cytokines production [8]. Some studies have reported that methylprednisolone reduces ventilation, respiratory shock, and mortality rate in ALI patients by improving the systemic inflammatory response [9]. However, the efficacy of methylprednisolone, one of the representative corticosteroids, is controversial, because some studies reported no significant benefit on ALI in clinical trials [10, 11]. In addition, there were unexpected side effects of corticosteroids such as hyperglycemia, hypertension, and further severe neuronal disorder [12, 13]. Commonly, inhalation of oxygen and restriction of intravenously received fluids are primary treatments for ALI; however, they may lead to pulmonary edema and interstitial pneumonitis due to the increase of pulmonary venous oxygenation caused by hyperoxia [14, 15]. Novel treatment derived from natural products with no side effects is required to treat ALI.

*Sosiho*-tang, known as Sho-saiko-to in Japanese and Xiao-Chai-Hu-Tang in Chinese, is an herbal medicine in Korea for medical insurance. *Sosiho*-tang consists of seven herbs: *Bupleurum falcatum* Linne 12 g, *Scutellaria baicalensis* Georgi 8 g, *Pinellia ternata* Breitenbach 4 g, *Panax ginseng* C. A. Meyer 4 g, *Glycyrrhiza glabra* Linn var. *glandulifera* Regel & Herder 2 g, 3 of *Zingiber officinale* Roscoe, and 2 of *Ziziphus vulgaris* Lamarck var. *inermis* Bunge. It is widely used for treating allergic diseases, cold-related symptoms, and chronic liver disease [16–19]. In particular, diseases for which medicine is efficacious of *Sosiho*-tang include the dyshepatia and hepatosis of chronic liver disorders [20, 21]. In the preclinical studies, *Sosiho*-tang improved liver inflammation and fibrosis by regulating liver toxicity levels, demonstrating that research of *Sosiho*-tang is focused on liver disease [19, 22, 23]. Because *Sosiho*-tang is reported to decrease the production of T helper 2-type cytokines and chemokine [24], we postulated that *Sosiho*-tang has a therapeutic effect on ALI via its anti-inflammatory effects. In the present study, inflammatory effects of *Sosiho*-tang against ALI were determined in LPS-sensitized mice and raw 264.7 cells.

## 2. Materials and Methods

### 2.1. *Sosiho*-Tang Soft Extract Sample

The sample of *Sosiho*-tang used in this study was Jungwoo Pharmacy *Sosiho*-tang Soft Ext. (SSHT), a prescription-based medicine in Korea. SSHT, a Korean Medicine for national health insurance, was obtained from Jungwoo Pharmacy Co., Ltd. (Lot. no. 702). Briefly, SSHT consists of 750 mg of *Bupleurum falcatum* Linne, 1.115 g of *Scutellaria baicalensis* Georgi, 588 mg of *Pinellia ternate* Breitenbach, 514 mg of *Panax ginseng* C. A. Meyer, 246 mg of *Glycyrrhiza glabra* Linn var. *glandulifera* Regel & Herder, 75 mg of *Zingiber officinale* Roscoe, and 518 mg of *Ziziphus vulgaris* Lamarck var. *inermis* Bunge soft extracts based on the general requirements in the Korean Pharmacopoeia. A total of 9 g of SSHT was dissolved in distilled water with appropriate doses for mice.

### 2.2. Animal Treatment

Thirty-six males C57BL/6 mice at 5 weeks of age were purchased from Raon Bio Inc. (Yongin, Korea). Mice were housed under controlled conditions (at 24 ± 2°C, relative humidity of 50–80%, and 12 h light/dark cycle). After 1-week acclimation, mice were randomly divided into six groups with 6 per group: CTR (nontreated group as a normal control), LPS (LPS-sensitized group as a negative control), DEX (LPS-sensitized and DEX-treated group as a positive control), S0.05 (LPS-sensitized and SSHT 0.05 g/kg-treated group), S0.55 (LPS-sensitized and SSHT 0.55 g/kg-treated group), and S5.55 (LPS-sensitized and SSHT 5.55 g/kg-treated group). 7 mg/kg of dexamethasone (DEX) and SSHT were orally preadministrated for 1 week, followed by intraperitoneal injection of 10 mg/kg LPS. The SSHT that was used for the human being 9 g/60 kg/day was converted by using the human equivalent dose (HED) equation used for mice into 5.55 g/kg. There was no toxicity to mice during sample administration of SSHT 5.55 g/kg. Following LPS sensitization for 2 h, blood samples were collected by cardiac puncture. Lung tissues were collected at 10% neutralized formalin solution. All experiments were approved by the Committee on Care and Use of Laboratory Animals of Kyung Hee University (KHUASP(SE)-18-078; Seoul, Korea).

### 2.3. Histological Analysis

The right inferior lobe of lung was fixed at 10% neutralized formalin for 24 h. The lung tissues were dehydrated and embedded in paraffin for 24 h. The paraffin blocks were cross cut into 4 *μ*m thickness and mounted on slides. These slides were stained with hematoxylin and eosin solution. The stained slides were photographed by digital microscope at a magnification of 200× and 400×. The blind test for ALI score was determined as 0 to 5 grades: 0, no injury and appears normal; 1, minimal (injury up to 25% of the field); 2, mild (injury between 25 and 50% of the field); 3, moderate (injury between 50 and 75% of the field); and 4, severe (>75%, diffuse injury). Tissue sections were examined by a pathologist blinded to the experiment.

### 2.4. Cell Treatment

Raw 264.7 cells were grown in Dulbecco's modified Eagle's medium (DMEM) (Gibco; Thermo Fisher Scientific, Inc., Waltham, MA, USA) supplemented with 10% v/v fetal bovine serum (Gibco), 2 mM glutamine, 100 IU/ml penicillin, and 100 *μ*g/mL streptomycin (Gibco). Cells were grown at 37°C in an atmosphere containing 5% CO_2_ of 95% humidity. Cells were seeded in 6-well plates and treated with 1, 10, 100, 250, 500, and 1000 *μ*g/mL of SSHT in the presence of LPS 1 *μ*g/mL for 24 h. DEX was treated at the 1 *μ*g/mL concentration to raw 264.7 cells for 24 h.

### 2.5. Preparations of Protein Extracts

Whole left lobe of lung tissues containing the left-upper lobe and the lower lobe was homogenized using a radioimmunoprecipitation assay (RIPA) lysis buffer (50 mm Tris-HCl, pH 7.4, 1% Nonidet P-40, 0.5% sodium deoxycholate, and 150 mM NaCl) containing protease inhibitor cocktail (Roche Diagnostics, Indianapolis, IN, USA) for extracts of whole proteins. The lysed proteins were centrifuged at 15,928 g for 10 min at 4°C and collected supernatant was used to determine mitogen-activated protein (MAP) Kinase, ERK1/2, SAPK/JNK, and p38. To extract cytoplasmic proteins, frozen lung tissues were homogenized with cytoplasmic buffer (10 mM HEPES, pH 7.9, 20 mM KCl, 0.1 mM EDTA, 0.1 mM EGTA, 1 mM DTT, 0.15% Nonidet P-40, 50 mM *ß*-glycerophosphate, 10 mM NaF, and 5 mM Na3VO4) containing the protease inhibitor cocktail and centrifuged at 24 g for 5 min. The supernatants obtained from the homogenate are used to analyze I*κ*B-*α* and phospho-I*κ*B-*α*. For nuclear protein extraction, a nuclear lysis buffer (20 mM HEPES, pH 7.9, 400 mM NaCl, 1 mM EDTA, 1 mM EGTA, 1 mM DTT, 0.5% Nonidet P-40, 50 mM *ß*-glycerophosphate, 10 mM NaF, and 5 mM Na3VO4) containing a protease inhibitor cocktail was added to the pellet. Nuclear protein was centrifuged at 15,928 g for 10 min to detect NF-*κ*B.

### 2.6. Western Blotting Analysis

The amount of proteins was quantified using the Bradford protein assay. Protein samples (20 *μ*g/lane) were separated into 10% SDS-PAGE gel and transferred to polyvinylidene fluoride (PVDF) membranes. The membrane was blocked for 1 h in TBS containing 0.1% Tween (TBS-T) and 5% bovine serum albumin. The membrane was incubated overnight at 4°C with primary antibodies. The following antibodies were used in this study: *ß*-actin (1:1000; cat. no. sc-47778; Santa Cruz Biotechnology), ERK1/2 (1:1000; cat. no. 4695S; Cell Signaling Technology), phospho-ERK1/2 (1:1000; cat. no. 4370S; Cell Signaling Technology), SAPK/JNK (1:1000; cat. no. 9252S; Cell Signaling Technology), phospho-SAPK/JNK (1:1000; cat. no. sc-293138; Santa Cruz Biotechnology), p38 (1:1000; cat. no. 9212; Cell Signaling Technology), phospho-p38 (1:1000; cat. no. sc-166182; Santa Cruz Biotechnology), NF-*κ*B (1:1000; cat. no. 3034S; Cell Signaling Technology), I*κ*B-*α* (1:1000; cat. no. sc-1643; Santa Cruz Biotechnology), and phospho-I*κ*B-*α* (1:1000; cat. no. sc-8404; Santa Cruz Biotechnology). Anti-mouse IgG (1:3000; cat. no. sc-516102; Santa Cruz Biotechnology) and anti-rabbit IgG (1:4000; cat. no. sc-2357; Santa Cruz Biotechnology) secondary antibodies were incubated room temperature for 1 h. The blots were visualized by Davinch-Chemi (cat no. CAS400 MF; Davinch-K, Seoul, Korea) with enhanced chemiluminescence kit (cat. no. ABC-3001; AbClon, Seoul, Korea).

### 2.7. Reverse Transcription Polymerase Chain Reaction (RT-PCR) Analysis

For RNA extraction, the lung tissues and raw 264.7 cells were homogenized in TRIzol reagent (Invitrogen Corp., Carlsbad, CA, USA), according to the manufacturer's instructions. The optical density was read at a wavelength of 260 nm to quantify RNA. To synthesize complementary DNA, Maxime RT PreMix (Invitrogen) was mixed with 1 *μ*g RNA at 45°C for 1 hour and incubated at 95°C for 5 min. PCR was performed using Maxime PCR PreMix (Invitrogen) and synthesized cDNA template. The sequence of the primers used was as shown in Table 1. The amplification conditions were 94°C for 30 sec, followed by 40 cycles of 94°C for 1 min, range from 58 to 70°C for 30 sec, 72°C for 1 min and final extension at 72°C for 5 min. The relative expressions of gene were calculated and normalized to GAPDH and analyzed by ImageJ software.

### 2.8. Statistical Analysis

Significance was determined by one-way analysis of variance (ANOVA) and Tukey's multiple comparison tests. In all analyses, *p* < 0.05 was taken to indicate statistical significance.

## 3. Results

### 3.1. Effects of SSHT on the Histological Analysis of Lung Tissue

**Figure 1:**
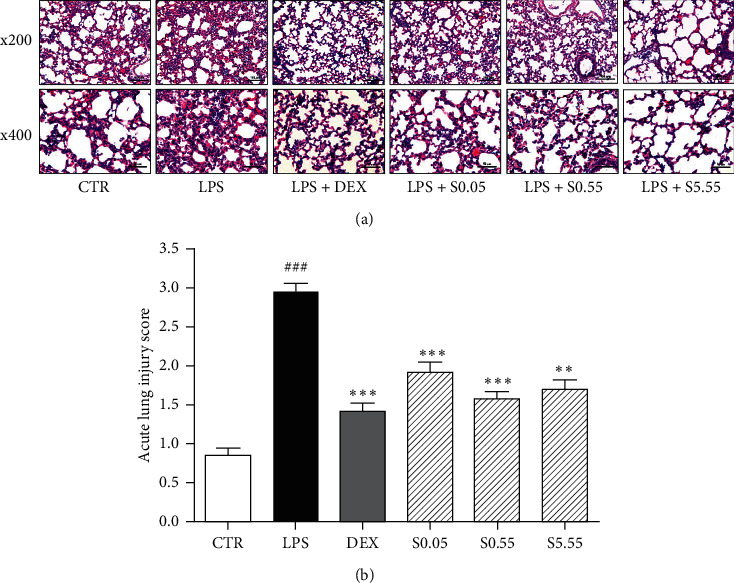
Histological changes of lung tissues indicated by hematoxylin and eosin staining. Representative images of lung tissues (a) and ALI score (b). The sections were stained with hematoxylin and eosin (H&E). Original magnifications were 200× and 400×. CTR: normal control group; LPS: LPS-sensitized ALI group; DEX: DEX-treated and LPS-sensitized group; S0.05: 0.05 g/kg SSHT-treated and LPS-sensitized group; S0.55: 0.55 g/kg SSHT-treated and LPS-sensitized group; and S5.55: 5.55 g/kg SSHT-treated and LPS-sensitized group. Results are presented as mean ± S.E.M.  ^###^*p* < 0.001 compared with CTR group;  ^*∗∗∗*^*p* < 0.001 and  ^*∗∗*^*p* < 0.01 compared with the LPS group.

Histological analysis was conducted to evaluate whether SSHT recovers LPS-induced change of lung structure. Compared to the structure of lung tissues in normal group, the LPS group led to alveolar hemorrhage, alveolar wall thickening, and alveolar spaces shrinkage. In contrast, SSHT treatment relieved alveolar wall thickness and alveolar spaces shrinkage similar to normal group (Figure 1(a)). The ALI score by LPS sensitization was 3.5 times higher than nontreated CTR group (*p* < 0.05). SSHT treatment at all concentrations (0.05, 0.55, and 5.55 g/kg) significantly decreased the histology scores by 34.5%, 46.2%, and 42.2% (*p* < 0.05), respectively, while DEX treatment showed 51.8% reduction (*p* < 0.05) of ALI score in LPS-sensitized mice (Figure 1(b)).

### 3.2. Effects of SSHT on Expression of Inflammatory Cytokines IL-6, TNF-*α*, and IFN-*γ* in Lung Tissues and Raw 264.7 Cells

**Figure 2:**
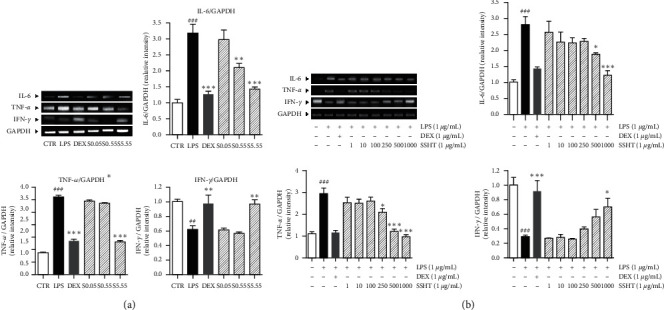
Expressions of cytokines in lung tissues (a) and raw 264.7 cells (b). CTR: normal control group; LPS: LPS-sensitized ALI group; DEX: DEX-treated and LPS-sensitized group; S0.05: 0.05 g/kg SSHT-treated and LPS-sensitized group; S0.55: 0.55 g/kg SSHT-treated and LPS-sensitized group; and S5.55: 5.55 g/kg SSHT-treated and LPS-sensitized group. Results are presented as mean ± S.E.M.  ^###^*p* < 0.001 and  ^##^*p* < 0.01 compared with CTR group and nontreated cells;  ^*∗∗∗*^*p* < 0.001,  ^*∗∗*^*p* < 0.01, and  ^*∗*^*p* < 0.05 compared with the LPS group and LPS-sensitized cells.

To examine whether SSHT treatment inhibits LPS-induced inflammation in ALI model, proinflammatory cytokines IL-6, TNF-*α*, and interferon-*γ* (IFN-*γ*) in lung tissue was assessed by RT-PCR. Expression of IL-6 was increased 3.18 times (*p* < 0.05) in LPS-treated group compared to the normal group. SSHT treatment at the concentrations of 0.55 and 5.55 g/kg decreased expression of IL-6 to 34.1% and 55.4% of LPS-treated group, respectively (*p* < 0.05). The levels of TNF-*α* increased 3.56 times of LPS-treated group than that in the normal group (*p* < 0.05). Expression of TNF-*α* was decreased 58.5% in treated with 5.55 g/kg of SSHT (*p* < 0.05). In contrast with the other cytokine expression levels, expression of IFN-*γ* was 0.57 times decreased in LPS-treated group compared with the nontreated CTR group (*p* < 0.05). SSHT treatment with 5.55 g/kg increased the expression of IFN-*γ* 35.94% compared to LPS group (Figure 2(a)) (*p* < 0.05).

Additionally, the expressions of IL-6 and TNF-*α* in LPS-sensitized raw 264.7 cells were 2.8 and 3.0 times increased compared to nontreated cells (*p* < 0.05), while LPS significantly decreased the mRNA level of IFN-*γ* in raw 264.7 cells (*p* < 0.05). In the SSHT-treated cells in the presence of LPS, the expressions of IL-6 and TNF-*α* were markedly decreased. 500 and 1000 *μ*g/mL of SSHT significantly inhibited the IL-6 levels, while 250, 500, and 1000 *μ*g/mL of SSHT significantly reduced the TNF-*α* levels, respectively (*p* < 0.05). Moreover, 1000 *μ*g/mL of SSHT significantly increased the IFN-*γ* expression in LPS-sensitized raw 264.7 cells (Figure 2(b)) (*p* < 0.05).

### 3.3. Effects of SSHT on NF-*κ*B Translocation and I*κ*B-*α* Phosphorylation in Lung Tissue

**Figure 3:**
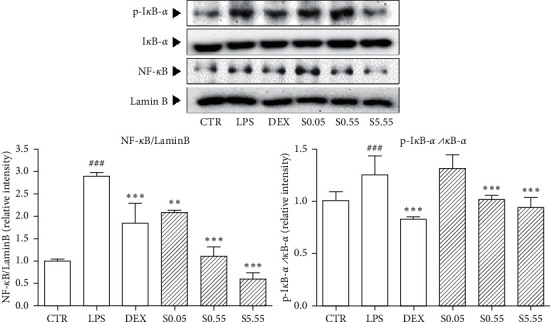
Expression of nuclear NF-*κ*B and cytoplasmic I*κ*B-*α* of lung tissues indicated by western blot analysis. CTR: normal control group; LPS: LPS-sensitized ALI group; DEX: DEX-treated and LPS-sensitized group; S0.05: 0.05 g/kg SSHT-treated and LPS-sensitized group; S0.55: 0.55 g/kg SSHT-treated and LPS-sensitized group; and S5.55: 5.55 g/kg SSHT-treated and LPS-sensitized group. Results are presented as mean ± S.E.M.  ^###^*p* < 0.001 compared with the CTR group;  ^*∗∗∗*^*p* < 0.001 and  ^*∗∗*^*p* < 0.01 compared with the LPS group.

I*κ*B-*α*/NF-*κ*B pathway is a key regulator of inflammation and highly activated in ALI, while LPS treatment induced the increases of 1.24 times on phosphorylated I*κ*B-*α* and 1.66 times on translocated NF-*κ*B, respectively (*p* < 0.05). I*κ*B-*α* phosphorylation was reduced 18.6% and 24.3% by 0.55 and 5.55 g/kg of SSHT treatment, respectively (Figure 3) (*p* < 0.05). Moreover, expression of nuclear NF-*κ*B was also decreased 63.5%, 67.9%, and 88.9% by SSHT treatment (*p* < 0.05).

### 3.4. Effects of SSHT on Expression of MAP Kinase in Lung Tissue

**Figure 4:**
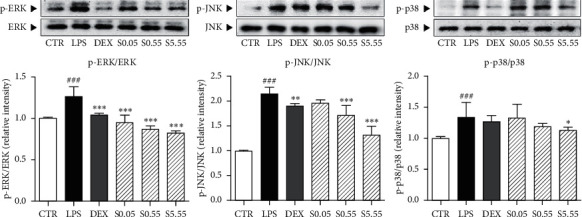
Expressions of MAP kinase of lung tissues indicated by western blot analysis. CTR: normal control group; LPS: LPS-sensitized ALI group; DEX: DEX-treated and LPS-sensitized group; S0.05: 0.05 g/kg SSHT-treated and LPS-sensitized group; S0.55: 0.55 g/kg SSHT-treated and LPS-sensitized group; and S5.55: 5.55 g/kg SSHT-treated and LPS-sensitized group. Results are presented as mean ± S.E.M.  ^###^*p* < 0.001 compared with the CTR group;  ^*∗∗∗*^*p* < 0.001,  ^*∗∗*^*p* < 0.01, and  ^*∗*^*p* < 0.05 compared with the LPS group.

LPS-induced 1.36 times increase of ERK phosphorylation in lung tissues of mice (*p* < 0.05). Administration of SSHT at all concentrations (0.05, 0.55, and 5.55 g/kg) had significantly dose-dependently decreased the ERK phosphorylation by 13%, 26.4%, and 56.8% (*p* < 0.05). Phosphorylation of JNK was significantly increased by 2.65 times in LPS-treated group compared with the CTR group (*p* < 0.05), whereas 0.55 and 5.55 g/kg of SSHT treatment markedly reduced LPS-induced phosphorylated JNK by 33.7% and 41% (*p* < 0.05). In LPS-sensitized mice, phosphorylation of p38 was 1.12 times increased in comparison with normal mice (*p* < 0.05). SSHT 5.55 g/kg-treated group showed a significant 15.7% reduction of p38 phosphorylation (*p* < 0.05) (Figure 4).

## 4. Discussion

New finding of efficacy of medicine would be helpful to expand its medical indication as a prescription for health insurance. Recommended daily intake dose of SSHT to human is 9 g/day/60 kg, converted to 5.55 g/kg of SSHT in mice. In this study, 0.05, 0.55, and 5.55 g/kg of SSHT were administered to LPS-induced ALI mice. LPS causes histological changes including alveolar hemorrhage, shrinkage of alveolar spaces, and alveolar wall thickening [25]. Those symptoms can be prevented by treatment with SSHT at a concentration of 5.55 g/kg.

*B*. *falcatum* Linne polysaccharides, a major herb of SSHT, suppressed LPS-induced proinflammatory cytokines production including IL-6, IL-1*β*, IFN-*β*, and TNF-*α* in peritoneal macrophages of mice [26]. Additionally, baicalin derived from *S. baicalensis* Georgi consisting of SSHT has been studied to decrease TNF-*α* and IL-1*β* levels in bronchoalveolar lavage fluids of LPS-induced ALI mice [27]. Total saponin from Ginseng as another major herb of SSHT was reported to inhibit LPS-induced increase of serum TNF-*α* level [28]. Based on the previous evidence, we anticipated that SSHT has inhibitory effects against ALI by regulating proinflammatory cytokines. In particular, lung proinflammatory cytokine expressions such as IL-6, TNF-*α*, and IFN-*γ* in ALI model were investigated to find the underlying mechanism of SSHT on the recovery of alveolar wall thickening. Proinflammatory cytokines such as IL-6 and TNF-*α* are considered key components of the acute inflammatory response [29–31]. Some studies have shown that TNF-*α* produced by initiating inflammatory response in macrophages undergo to activate NF-*κ*B pathway and MAP kinase [32]. IL-6 is a multifunctional cytokine produced by monocytes and macrophages in the LPS-induced immune response [33]. IFN-*γ* as a primary activator by stimulating innate and adaptive immunity clears intracellular pathogens and produces enzymes to inhibit viral infections [34, 35]. IL-6 and TNF-*α* mRNA levels in the lung tissues and raw 264.7 cells were increased by exposure of LPS and significantly decreased by the pretreatment or cotreatment with SSHT. Those results demonstrate that SSHT might regulate the counterbalance of inflammatory responses by downregulating IL-6 and TNF-*α* and upregulating IFN-*γ*.

**Figure 5:**
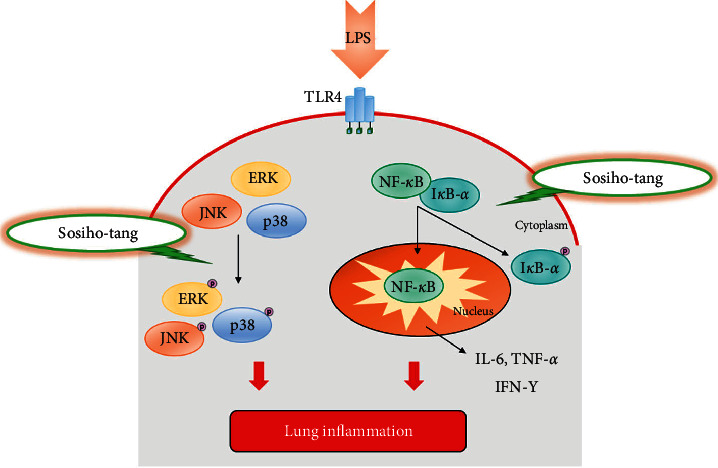
Schematic diagram of potential action of SSHT on lung inflammation. SSHT appears to regulate the IL-6, TNF-*α*, and IFN-*γ*, following the inhibition of NF-*κ*B and MAP kinases expressions. These actions inhibit the LPS-induced lung inflammation in ALI.

NF-*κ*B is translocated from the cytoplasm to the nucleus by inflammatory cytokines such as IL-6 and TNF-*α* [36–40]. The nuclear translocation of NF-*κ*B is regulated by I*κ*B-*α* proteolytic degradation [41]. Additionally, MAP kinases including ERK12, SAPK/JNK, and p38 are associated with the production of cytokines in the inflammatory response [42]. Inhibition of NF-*κ*B and MAP kinases in LPS-sensitized lung tissues could ameliorate ALI [43]. The expression of NF-*κ*B and the phosphorylation of I*κ*B-*α* in LPS-sensitized lung tissue were increased, but they were markedly decreased in the SSHT-treated mice. SSHT also significantly reduced the phosphorylation of ERK1/2, SAPK/JNK, and p38 in the lungs. In addition to the results from IL-6 and TNF-*α*, SSHT attenuated the activation of MAP kinase and NF-*κ*B by inhibition of inflammatory cytokines such as IL-6 and TNF-*α* (Figure 5).

## 5. Conclusion

In conclusion, SSHT alleviated the inflammatory IL-6 and TNF-*α* production, following the decreases of transcription factors including NF-*κ*B and MAP kinases. Inhibition of inflammatory responses by SSHT in lung would be helpful to ameliorate LPS-induced ALI. Taken together, SSHT might be beneficial for treating ALI. Since the administration of 5.55 g/kg of SSHT to mice is converted to daily intake 9 g/day/60 kg dose for human, Jungwoo Pharmacy *Sosiho*-tang Soft Ext. is applicable for treating lung inflammation. Optimal dosage range of SSHT might be 9 g/day/60 kg, a daily intake dose for human in present. Further studies regarding the maximal efficacy and toxicity of SSHT are needed.

## Figures and Tables

**Table 1:** Sequence of reverse transcription PCR primers.

Target gene	5′ ⟶ 3′ forward primer	5′ ⟶ 3′ reverse primer
IL-6	CGGAGAGGAGACTTCACAGAGGA	GGAGAGCATTGGAAATTGGGG
TNF-*α*	CCTGTAGCCCACGTCGTAGC	TTGACCTCAGCGCTGAGTTG
IFN-*γ*	AGCGGCTGACTGAACTCAGATTGTAG	GTCACAGTTTTCAGCTGTATAGGG
GAPDH	GGCATGGACTGTGGTCATGA	TTCACCACCATGGAGAAGGC

## Data Availability

The data used to support the findings of this study are available from the corresponding author upon reasonable request.

## Abbreviations

ALI: Acute lung injury
HED: Human equivalent dose
IFN-*γ*: Interferon-*γ*
IL: Interleukin
LPS: Lipopolysaccharide
MAP: Mitogen-activated protein
SSHT: *Sosiho*-tang
TNF-*α*: Tumor necrosis factor-*α*.
